# Identification and functional characterisation of DNA methylation differences between East- and West-originating Finns

**DOI:** 10.1080/15592294.2024.2397297

**Published:** 2024-09-01

**Authors:** Joanna Ciantar, Saara Marttila, Sonja Rajić, Daria Kostiniuk, Pashupati P Mishra, Leo-Pekka Lyytikäinen, Nina Mononen, Marcus E Kleber, Winfried März, Mika Kähönen, Olli Raitakari, Terho Lehtimäki, Emma Raitoharju

**Affiliations:** a Molecular Epidemiology (MOLE), Faculty of Medicine and Health Technology, Tampere University, Tampere, Finland; b Gerontology Research Center, Tampere University, Tampere, Finland; c Tays Research Services, Wellbeing Services County of Pirkanmaa, Tampere University Hospital, Tampere, Finland; d Department of Clinical Chemistry, Tays Research Services, Fimlab Laboratories, and Finnish Cardiovascular Research Center, Faculty of Medicine and Health Technology, Tampere University, Tampere, Finland; e Vth Department of Medicine (Nephrology, Hypertensiology, Endocrinology, Diabetology, Rheumatology), Medical Faculty of Mannheim, Heidelberg University, Mannheim, Germany; f SYNLAB MVZ Humangenetik Mannheim, Mannheim, Germany; g Synlab Academy, SYNLAB Holding Deutschland GmbH, Mannheim, Germany; h Department of Clinical Physiology, Tampere University Hospital and Faculty of Medicine and Health Technology, Tampere University, Tampere, Finland; i Centre for Population Health Research, University of Turku and Turku University Hospital, Turku, Finland; j Research Centre of Applied and Preventive Cardiovascular Medicine, University of Turku, Turku, Finland; k Department of Clinical Physiology and Nuclear Medicine, Turku University Hospital, Turku, Finland; l Finnish Cardiovascular Research Center Tampere, Faculty of Medicine and Health Technology, Tampere University, Tampere, Finland; m Fimlab Laboratories, Tampere, Finland

**Keywords:** DNA methylation, East and West Finns, population studies, multi-omic analysis, EWAS, quantitative trait loci

## Abstract

Eastern and Western Finns show a striking difference in coronary heart disease-related mortality; genetics is a known contributor for this discrepancy. Here, we discuss the potential role of DNA methylation in mediating the discrepancy in cardiometabolic disease-risk phenotypes between the sub-populations. We used data from the Young Finns Study (*n* = 969) to compare the genome-wide DNA methylation levels of East- and West-originating Finns. We identified 21 differentially methylated loci (FDR < 0.05; Δβ >2.5%) and 7 regions (smoothed FDR < 0.05; CpGs ≥ 5). Methylation at all loci and regions associates with genetic variants (*p* < 5 × 10^−8^). Independently of genetics, methylation at 11 loci and 4 regions associates with transcript expression, including genes encoding zinc finger proteins. Similarly, methylation at 5 loci and 4 regions associates with cardiometabolic disease-risk phenotypes including triglycerides, glucose, cholesterol, as well as insulin treatment. This analysis was also performed in LURIC (*n* = 2371), a German cardiovascular patient cohort, and results replicated for the association of methylation at cg26740318 and DMR_11p15 with diabetes-related phenotypes and methylation at DMR_22q13 with triglyceride levels. Our results indicate that DNA methylation differences between East and West Finns may have a functional role in mediating the cardiometabolic disease discrepancy between the sub-populations.

## Introduction

**Figure 1.**
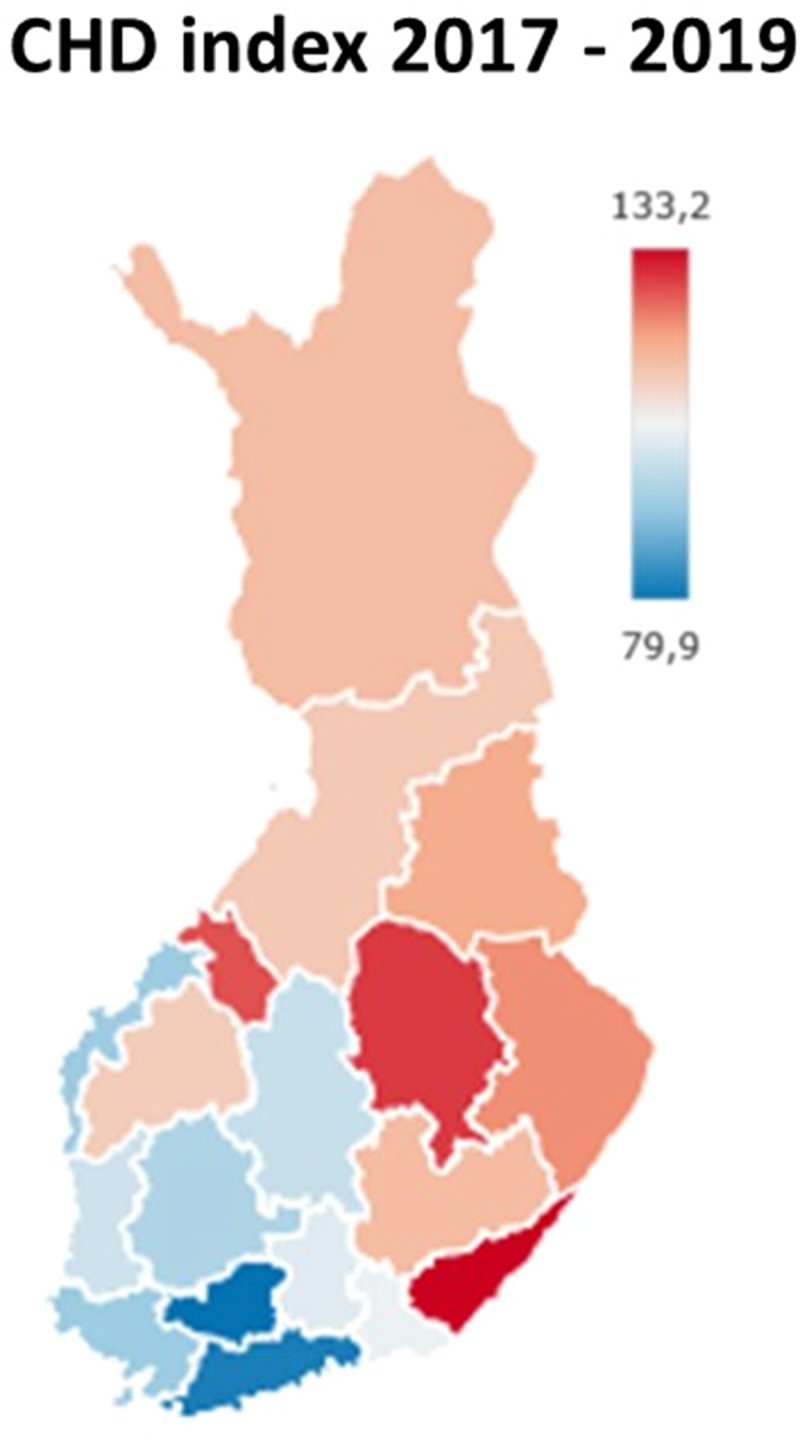
Age-standardized coronary heart disease index in Finland. Data from the Finnish Institute for Health and Welfare shows the distribution of coronary heart disease (CHD) in the country. The index is calculated relative to the national average which is given a value of 100. Regions with an age-adjusted CHD index greater than the national average are redder in colour while those with a lower value are bluer, on a continuous colour scale. Map generated using the THL morbidity index online tool [15].

The Finnish population exhibits a striking division between the Northeast and the Southwest regions, characterised by differences in lifestyle, dialects, genetics and disease prevalence, including coronary heart disease (CHD) [1–4]. The East–West discrepancy in CHD mortality was first documented in the 1940s-50s and led to the launching of The North Karelian Project in 1972, with the aim of reducing CHD mortality in East Finland [5]. Previous research from The Young Finns Study (YFS), a longitudinal cohort set up with the aim of investigating these regional differences, identified higher total cholesterol, lower HDL-cholesterol and higher triglycerides in East-originating individuals in their childhood and adolescence [6,7]. Higher levels of Lp(a), consistent across all time points, were also identified in Eastern Finns [8]. Nation-wide educational campaigns were successful in reducing CHD mortality across Finland [9,10]; however, a relatively elevated level of CHD mortality in Northeast Finland remains to this day [11,12] (Figure 1). This residual risk difference could be partially attributed to differences in the genetic background of the two sub-populations [13,14].

Since previous research on cardiometabolic disease (CMD) risk differences between Eastern and Western Finns has centred around lifestyle and genetics, we wanted to investigate the potential role of DNA methylation in this context. In mammals, DNA methylation predominantly occurs on cytosine bases that are linked to an adjacent guanine base via a phosphate, therefore termed CpGs [16]. The DNA methylation profile changes with normal ageing processes [17,18] and in several diseases including cancer [19,20], neurological diseases [21,22] and CMDs [23–26]. DNA methylation differences between healthy individuals can also arise due to environmental or lifestyle differences, such as smoking [27–29] and obesity [30,31], as well as genetic variants, known as methylation quantitative trait loci (meQTLs) [32,33]. Several studies have shown that genetic differences between populations significantly contribute to their different DNA methylation profiles [34,35]. The inheritance of meQTLs is a potential mechanism for the transmittance of cardiovascular disease risk across generations [36].

To investigate the potential role of DNA methylation in explaining the difference in CHD risk between Eastern and Western Finns, we utilised multi-omic data from the YFS cohort. We set out with three main aims: 1) to identify differentially methylated loci between East- and West-originating Finns 2) to identify meQTLs that might be regulating the differentially methylated loci and 3) to assess the role of DNA methylation at these loci in the regulation of gene expression and CMD-risk phenotypes. The LUdwigshafen RIsk and Cardiovascular Health (LURIC) cohort was used as a replication cohort for the associations between methylation and CMD risk factors.

## Materials and methods

### Study population: young finns study (YFS)

YFS is a longitudinal multi-omic population cohort launched in 1980 with the aim of investigating regional differences in CHD risk factors in Finland [37]. The study participants (*n* = 3,596), aged between 3 and 18 y, were recruited from five Finnish cities (Helsinki, Tampere, Turku, Oulu and Kuopio) and the nearby countryside. Study participants were sampled randomly from the Finnish national registry with an equal number of boys and girls and participants from Eastern and Western parts of the country. Follow-ups were performed every 3–9 y with the most recent one in 2018 which also included the parents and offspring of the original participants. Blood samples were taken by a uniform protocol at each of the five data collection sites by specially trained health professionals recruited for the study. The blood samples were frozen, transported to a centralised storage location and henceforth processed uniformly. Data utilized in this study was collected in the 2011 follow-up and includes DNA methylation, genotype, gene expression and biochemical blood measurements.

**Figure 2.**
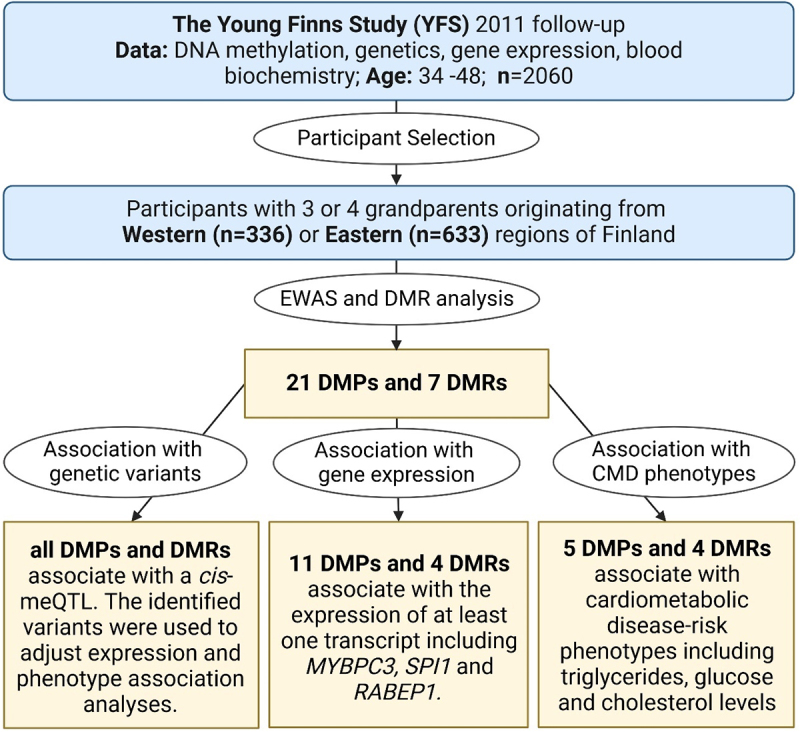
Flowchart of the study. Participants from the Young Finns Study (YFS) were selected to be included in this study based on their Eastern or Western origins, which were defined as having three or four grandparents originating from the same area. An epigenome-wide association study (EWAS) and Differentially Methylated Region (DMR) analysis revealed 21 differentially methylated positions (DMPs) and 7 DMRs between Eastern and Western originating Finns. Methylation at these loci was investigated for its association with underlying genetic variants, gene expression and cardiometabolic disease (CMD) risk phenotypes. The association between methylation at the identified loci and CMD risk phenotypes was replicated in LURIC, a German cardiovascular patient cohort (not shown in figure). Figure created with BioRender.com.

A subset of the YFS cohort, determined by the participant’s self-reported grandparental birthplaces and the availability of DNA methylation data, was used to answer the primary research question presented in this study (Figure 2). Participants with at least three grandparents originating from Eastern or Western Finland were selected (*n* = 969). A previously defined East–West demarcation [38] was used to categorize the Finnish regions.

### Replication cohort: the LUdwigshafen RIsk and Cardiovascular health (LURIC)

LURIC is a German cardiovascular patient cohort recruited between 1997 and 2000 [39]. The participants had undergone coronary angiography at the Ludwigshafen hospital and were eligible for the study if they did not have any acute or chronic diseases other than cardiac diseases. Ages of the participants varied from 19 to 91 (mean ages of male and female participants were 65 and 62, respectively) at the time of recruitment. In the present work, this cohort is used as a replication cohort for the association between DNA methylation and cardiometabolic phenotypes.

### DNA methylation data

For YFS, DNA was extracted from EDTA-blood samples using the Wizard Genomic DNA Purification Kit, following the manufacturer’s instructions. DNA methylation levels were measured using the Illumina Infinium MethylationEPIC BeadChip, following the Illumina protocol, for both cohorts. The array measures DNA methylation at approximately 850,000 CpG sites for a genome-wide coverage. The YFS samples from different collection sites were randomised on the arrays to avoid potential confounding batch effects.

DNA methylation data from YFS was pre-processed using the *minfi* R package [40] as previously described [41]. After preprocessing, 769,683 probes were retained, having excluded those which (i) target sites on sex chromosomes, (ii) are known to be cross-reactive or (iii) target known single nucleotide polymorphisms. Blood cell proportions were estimated with the Houseman method [42] using the *minfi* R package.

LURIC DNA methylation data was processed following the CPACOR pipeline [43] and has been described previously [44]. Briefly, probe exclusion was performed using the *DMRcate* R package [45] and 795,619 autosomal probes were retained. Methylation values were then quantile normalized. DNA methylation data from LURIC is available for 2371 participants.

### Genotyping

Genotyping of YFS participants was performed using a custom Illumina Human 670k BeadChip as described previously [46]. Genotype imputation was performed using TOPMed r2 as reference [47]. After QC checks, genotype data for 546,677 SNPs in 2443 individuals was retained. Genotyping in LURIC was performed with the Affymetrix Human SNP Array 6.0 [48]. After QC checks, genotype data for 700,714 SNPs in 2947 individuals was retained. Imputation was performed with the 1000 Genomes Phase I integrated variant set [49] or TOPMed r2 as reference. This study utilized genetic data from both imputations as indicated in Table S1.

### Gene expression profiling

Whole blood for gene expression profiling was collected from YFS participants in PaXgene Blood RNA Tubes and RNA was isolated using the PAXgene Blood microRNA Kit. Genome-wide gene expression data was measured from blood samples collected in 2011 with the Illumina HumanHT-we version 4 Expression BeadChip as described previously [50]. Raw expression data was exported from GenomeStudio and processed in R studio. Nonparametric background correction was performed, followed by quantile normalisation and log2 transformation using the *limma* R package [51].

### Biochemical blood measurements

Phenotype data for 2063 YFS participants and 3316 LURIC participants was collected using a combination of physical examinations and questionnaires. Fasting blood samples were also collected at this time and used to measure glucose, insulin, triglycerides, lipoprotein cholesterols and C-reactive protein by standard clinical assays as previously described for YFS [52] and for LURIC [39].

### Identification of DMPs between Eastern and Western Finns

Classification of ‘Eastern’ and ‘Western’ Finnish regions was performed as described previously [38]. An epigenome-wide association study (EWAS) was performed using linear regression in R to determine differentially methylated CpG sites between East- and West-originating Finns (Figure 2). The model was adjusting with age, sex, BMI, smoking status, methylation-based estimation of blood cell composition and the first 30 principal components of the control probes. To account for multiple testing, p-values were adjusted with False Discovery Rate (FDR) correction. Difference in methylation (Δβ) between the groups was calculated for each CpG site by subtracting the median value for the West-originating participants from the median value of the East-originating participants.

### Identification of DMRs between Eastern and Western Finns

Differentially methylated regions (DMRs) were identified using the *DMRcate* R package [45]. Beta methylation values were first converted to *M* values using the formula log2(β)/(1-β). M methylation values were then used to calculate DMRs with lambda = 1000, C = 2 and the same co-variates as described previously for EWAS. DMRs with a minimum of 5 CpG sites were retained for downstream analysis. The mean methylation level for each DMR was calculated for all participants, and this was used in downstream analyses of genetic, gene expression and phenotype association.

### Epigenetic clocks

Dunedin PACE epigenetic ages were calculated using the *DunedinPACE* R package [53], while principle component clocks and age acceleration residuals for Horvath, Hannum, Pheno Age and Grim Age [54] were calculated using the code provided by the authors. Inverse normal transformed epigenetic age residuals were compared between East- and West-originating Finns using regression models in R for all participants and separately for men and women. Regression models were adjusted with age, smoking and BMI.

### Genetic association studies

Genetic association studies were performed to identify *cis*-genetic variants (within 1Mb) that associate with DNA methylation (meQTL; Figure 2) and gene expression (eQTLs). All genetic association analyses were performed using PLINK 2.0 [55]. Statistically significant variants (p < 5e^−8^) with a minor allele frequency of ≥0.02 which passed the Hardy-Weinberg check (p ≥ 1e^−6^) and had less than 5% missingness were identified. When multiple variants met these criteria, the variant with the lowest *p* value was selected as the top-associating variant.

### Gene expression association studies

Association analysis between methylation at previously identified DMPs and DMRs between East- and West-originating Finns and gene expression was performed on gene transcripts within 100kb of the methylation locus/region (Figure 2). Target genes were identified using the *biomaRt* R package [56]. Association analyses were performed using three linear regression models, the first containing no covariates, the second containing only the respective associating meQTL as a covariate and the third containing both the respective associating meQTL and eQTL.

### Association with cmd-related risk phenotypes

The analysis of the association between methylation at DMPs and DMRs and cardiometabolic phenotypes was performed with regression models in R (Figure 2). All models were adjusted with sex, age, smoking status and BMI for both YFS and LURIC, as well as fasting status upon blood collection for YFS. Inverse normal transformation was applied to all continuous dependent variables to normalize the variable distribution.

The continuous phenotypes included in the analyses for YFS were mean systolic and diastolic blood pressure (mmHg), glucose (mmol/l), insulin (mmol/l), HbA1c (mmol/mol), total cholesterol (mmol/l), LDL cholesterol (mmol/l), HDL cholesterol (mmol/l), triglycerides (mmol/l), apolipoprotein A-I (g/l), apolipoprotein B (g/l) and lipoprotein (a) (mg/l). Categorical phenotypes were hypertension, hypertension medication, hypercholesterolemia medication, insulin therapy, oral treatment for diabetes, diabetes type I and II, metabolic syndrome by harmonizing definition [57], cerebrovascular disease, heart bypass surgery, angioplasty on heart’s blood vessels, heart failure, atrial fibrillation, other arrhythmia, congenital heart defect, cerebral thrombosis, cardiac infarction and coronary artery-induced chest pain.

The phenotypes included in the analyses for LURIC were mean systolic blood pressure (mmHg), mean diastolic blood pressure (mmHg), fasting blood glucose (mg/dL), fasting insulin (U/l), triglycerides (mg/dL), total cholesterol (mg/dL), LDL cholesterol (mg/dL), HDL cholesterol (mg/dL), HbA1c (%), lipoprotein (a) (mg/dL), Apolipoprotein B (mg/dL), myocardial infarction, history of hypertension, diabetes type 2, insulin treatment and venous thrombosis.

## Results

### The study population

In this study, we used a subset of the YFS (*n* = 969) which included Finnish participants with Eastern or Western origins based on self-reported grandparental birthplace (Figure 2). Around half of the participants with Eastern origins were born and were also living in Eastern regions in 2011, while the rest migrated to the West. In contrast, almost all of the participants with Western origins were born and were living in the West in 2011 (Fig S1). The two groups show comparable demographics (Table S2).

**Figure 3.**
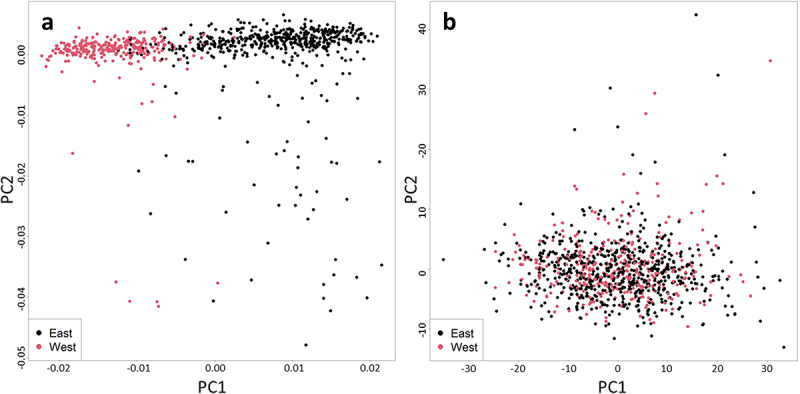
Variation in genetic and DNA methylation data of study participants. Scatter plots of the first two principal components of the genetic (A) and DNA methylation (B) data of the study cohort reveal a distinct clustering of East- and West-originating Finns only in the genetic data.

In order to validate our classification of East/West origins, we analysed the first two principal components of our genetic data (available for 822 out of the 969 participants included in this analysis). As expected, the greatest source of variation in the genetic data (PC1) is explained by the East/West origin of the participants (Mann-Whitney U-test p-value <2.2 × 10^−16^), evident by the clustering of the two groups (Figure 3a). A similar analysis of principal components of DNA methylation data, however, did not show similar clustering (Figure 3b), indicating that the East/West origins of participants was not among the top causes of DNA methylation variation in 2011 (Mann-Whitney U-test p-value for PC1 = 0.838 and PC2 = 0.13).

### DNA methylation differences between East- and West-originating Finns

To identify DMPs between East- and West-originating Finns, we performed an epigenome-wide association study (EWAS). The analysis revealed 82 statistically significant DMPs at FDR < 0.05 (Table S3). Of these, 21 CpGs had a difference in median methylation β value of ≥2.5% between the two groups (Table 1), which increases the potential biological relevance of these loci. These CpG sites were therefore selected for further analysis. Eastern Finns had higher methylation levels at 14 of these CpG sites, while Western Finns had higher levels in 7 CpG sites. Most CpG sites are located in inter-genic regions, while nine sites are located in gene bodies and four are in transcription start sites. Several DMPs identified between Eastern and Western Finns exhibit notable clustering, particularly in chromosomes 2 and 17.

**Table 1.** The 21 DMPs between East- and West-originating Finns.

CpG	Chr	Locus	Gene Name	Relation to Gene	Beta	SE	P value	FDR	Δβ
cg08946995	1	229283674	/	/	−0.03	0.006	1.3×10^−6^	3.1×10^−2^	5.1%
cg15720821	1	229284563	/	/	−0.03	0.007	8.7×10^−7^	2.7×10^−2^	3.5%
cg13902335	2	216876969	*MREG*	Body	0.02	0.004	1.5×10^−7^	1.4×10^−2^	−3.1%
cg10833299	2	242749776	*NEU4*	TSS1500	−0.05	0.008	4.9×10^−9^	9.3×10^−4^	8.1%
cg15470645	2	242749789	*NEU4*	TSS1500	−0.04	0.006	1.0×10^−10^	7.9×10^−5^	5.5%
cg26207766	2	242763794	/	/	−0.06	0.011	1.0×10^−7^	1.3×10^−2^	5.8%
cg03455424	2	242763982	/	/	−0.04	0.007	1.6×10^−9^	5.6×10^−4^	7.3%
cg18948877	2	242904738	*LINC01237*	Body	−0.02	0.004	3.0×10^−7^	1.9×10^−2^	2.5%
cg03514530	3	145525915	/	/	0.04	0.009	3.5×10^−6^	4.4×10^−2^	−6.4%
cg10619644	7	69149951	*AUTS2*	Body	0.03	0.006	1.9×10^−6^	3.2×10^−2^	−3.3%
cg26740318	11	18477153	*LDHAL6A*	TSS1500	−0.03	0.006	4.0×10^−6^	4.5×10^−2^	3.6%
cg00674440	11	47339067	*MADD*	Body	0.05	0.009	5.8×10^−7^	2.5×10^−2^	−8.6%
cg11472901	13	110527539	/	/	0.02	0.005	2.9×10^−6^	3.8×10^−2^	−3.0%
cg27489373	16	1507959	*CLCN7*	Body	−0.03	0.007	5.3×10^−6^	5.0×10^−2^	2.8%
cg23194629	17	5290760	*NUP88*	Body	−0.02	0.005	3.6×10^−6^	4.4×10^−2^	3.1%
cg01199135	17	71471080	*SDK2*	Body	−0.02	0.004	7.5×10^−8^	1.2×10^−2^	3.0%
cg09786634	17	78912393	*RPTOR*	Body	0.03	0.006	5.8×10^−7^	2.5×10^−2^	−3.0%
cg11949518	17	78912765	*RPTOR*	Body	0.06	0.012	4.2×10^−6^	4.7×10^−2^	−3.1%
cg00698263	17	78962984	/	/	−0.02	0.005	4.5×10^−6^	4.8×10^−2^	3.9%
cg03953789	17	78963035	/	/	−0.02	0.005	4.6×10^−6^	4.8×10^−2^	6.6%
cg14750269	19	46975180	*PNMAL1*	TSS1500	−0.02	0.004	8.8×10^−7^	2.7×10^−2^	3.6%

Significant DMPs (FDR <0.05 and Δβ ≥2.5%) between East- and West-originating Finns. Δβ is the difference in median methylation β value between the two groups (β_East_ - β_West_). The Beta column represents the regression coefficient. Body – CpG site located within the gene body; TSS1500 – CpG site located within 1500bp of the transcription start site; Shore – CpG site is located up to 2 kb from CpG island; Shelf – CpG site is located between 2 and 4 kb from CpG island. The table is sorted by genomic locations (GRCh37 build).

In addition to DMPs, we also wanted to identify larger differentially methylated regions (DMRs) as these may be more biologically relevant than individual CpG sites [58]. We identified seven DMRs (≥5 CpGs per region) between East- and West-originating Finns (Table 2). The regions span from 325 to 924 basepairs in length and contain up to 21 CpG sites. For the scope of this article, the DMRs were named according to the chromosome band in which they are located. DMR_2q37 overlaps with two DMPs (cg10833299 and cg15470645), while the DMR_11p15 overlaps with one DMP (cg26740318) that were identified in the EWAS. Eastern Finns are hypermethylated for all DMRs when compared to Western Finns (Fig S2), a trend which follows the EWAS results.

**Table 2.** Differentially methylated regions between Eastern and Western Finns.

Chr band	Start Position	End Position	Width	No. Of CpGs	Overlapping Genes	Smoothed FDR	Mean Δβ	Max Δβ
2q37	242749533	242749925	393	5	*NEU4, AC114730.3*	1.4×10^−14^	2.9%	8.2%
6p22	29648161	29649084	924	21	*ZFP57*	5.3×10^−23^	1.7%	5.1%
6p21	32121113	32121566	454	19	*PPT2, PRRT1*	8.5×10^−17^	0.5%	1.2%
8p21	22132563	22133356	794	13	*PIWIL2*	8.5×10^−17^	0.7%	2.4%
11p15	18477153	18477534	382	5	*LDHAL6A*	3.1×10^−14^	1.7%	3.6%
19q13	58220494	58220818	325	8	*ZNF551, AC003006.7, ZNF154*	8.9×10^−14^	0.8%	1.7%
22q13	38092719	38093207	489	10	*NOL12, TRIOBP*	5.3×10^−14^	0.7%	1.2%

DMR analysis revealed seven DMRs, containing ≥5 CpGs, between East- and West-originating Finns. Δβ is the difference in median methylation β value between the two groups (β_East_ - β_West_). Start and End positions for the DMR are based on the GRCh37 build.

### Global methylation and epigenetic age analysis

Next, we investigated whether there are differences in global methylation and epigenetic ages between East- and West-originating Finns. There was no statistically significant difference in global methylation between the two groups (*p* = 0.12; Fig S3). Epigenetic ages are an indicator of the biological health status and were calculated for DunedinPACE [53], a measure of the pace of aging, and PC clocks for Horvath, Hannum, PhenoAge and GrimAge [54]. Whole population and sex-stratified analysis revealed a statistically significant difference only in DunedinPACE ages of men, where East-originating men had a median value which was 0.02 higher than West-originating men (*p* = 0.014; Table S4).

### The genetic basis of DMPs and DMRs between East- and West-originating Finns

Since DNA methylation levels can be regulated both by genetic and environmental factors, we investigated whether methylation at each of the 21 identified DMPs associates with genetic variants within 1Mb of the locus. Multiple *cis*-meQTLs were identified for each CpG site (*p* < 5e^−8^; Table S5) and the variant with the lowest p-value was selected for further analysis. Similarly, mean methylation level of each DMR is associated with multiple genetic variants.

The strong associations of DMP and DMR methylation levels with genetics raised the question whether the observed DNA methylation differences between Eastern and Western Finns are due to an unequal distribution of genetic variants. To investigate this, we replicated the initial EWAS setting for the 21 DMPs with the addition of the respective associating top SNP as a covariate. The top SNP was considered to account for the genetic effect since there is high association between proximal SNPs due to linkage disequilibrium. Our results show a decrease in overall statistical significance when compared to the original EWAS setting (Table 3). However, the methylation at 13 CpG sites remained nominally significant after adjusting with the respective SNP, indicating that differential DNA methylation at these sites is not purely dependent on the underlying genetics, and other factors – such as environmental factors – could be involved. Interestingly, the clustering CpG sites on chromosome 2 all retained statistical significance while those on chromosome 17 did not.

**Table 3.** Adjusting the EWAS analysis with the top-associating *cis-*meQTL.

CpG	Chr	Locus	Gene Name	Top SNP	Beta	SE	P value
cg08946995	1	229283674	/	rs11583419	<0.001	0.002	8.6×10^−1^
cg15720821	1	229284563	/	rs72766103	−0.008	0.004	8.6×10^−2^
cg13902335	2	216876969	*MREG*	rs13013441	0.007	0.003	**2.4×10^−2^**
cg10833299	2	242749776	*NEU4*	rs4973673	−0.032	0.008	**8.4×10^−5^**
cg15470645	2	242749789	*NEU4*	rs34334574	−0.029	0.006	**1.1×10^−5^**
cg26207766	2	242763794	/	rs34334574	−0.038	0.012	**1.0×10^−3^**
cg03455424	2	242763982	/	rs34334574	−0.030	0.007	**5.7×10^−5^**
cg18948877	2	242904738	*LINC01237*	rs4973673	−0.009	0.004	**2.4×10^−2^**
cg03514530	3	145525915	/	rs7643638	0.018	0.006	**2.8×10^−3^**
cg10619644	7	69149951	*AUTS2*	rs56302473	0.008	0.003	**1.7×10^−2^**
cg26740318	11	18477153	*LDHAL6A*	rs34109624	−0.014	0.007	**3.5×10^−2^**
cg00674440	11	47339067	*MADD*	rs7947269	0.008	0.003	**2.2×10^−2^**
cg11472901	13	110527539	/	rs12583851	0.016	0.005	**3.9×10^−3^**
cg27489373	16	1507959	*CLCN7*	rs9941109	−0.022	0.007	**2.3×10^−3^**
cg23194629	17	5290760	*NUP88*	rs60379759	0.002	0.002	3.1×10^−1^
cg01199135	17	71471080	*SDK2*	rs11651551	−0.016	0.004	**2.0×10^−4^**
cg09786634	17	78912393	*RPTOR*	rs7219318	0.004	0.003	2.5×10^−1^
cg11949518	17	78912765	*RPTOR*	rs56239682	0.010	0.006	7.8×10^−2^
cg00698263	17	78962984	/	rs6565507	−0.003	0.003	3.8×10^−1^
cg03953789	17	78963035	/	rs1555643536	−0.002	0.003	5.6×10^−1^
cg14750269	19	46975180	*PNMAL1*	rs11083832	−0.003	0.002	2.3×10^−1^

A recreation of the original EWAS setting with the addition of the respective top SNP as a covariate resulted in 13 CpGs which retained nominal statistical significance (in bold). The chromosome, locus (GRCh37 build) and gene name pertain to the CpG site while the Top SNP column lists the rsID of the SNP added as a covariate.

### Association of methylation at DMPs and DMRs with proximal gene expression

Next, we investigated whether DNA methylation at the DMPs associates with the expression of transcripts located 100kb upstream and downstream of the methylation locus. We found significant (*p* < 0.001) associations between 13 DMPs and transcripts from 17 genes (Table S6). To account for the genetic effect on DNA methylation, we repeated the analysis and added the respective meQTL as a covariate. The association between 10 CpG sites and transcripts from 12 genes retained nominal statistical significance (Table 4A). We also made a third model which accounts for the effect of eQTLs, genetic variants that associate with gene expression, in addition to meQTL. A nominally significant association was retained for all previously identified associations except that of cg11949518 and *RPTOR* expression, and cg01199135 and *SDK2* expression. These results suggest that DNA methylation at the sites which retained statistical significance is likely to be mechanistically involved in gene expression regulation and not a bystander.

**Table 4.** Association of methylation at DMPs and DMRs with proximal gene expression.

A	CpG	Gene	Model 1: No covariates		Model 2: meQTL		Model 3: meQTL + eQTL	
			Beta	P value	Beta	P value	Beta	P value
	cg00674440	*DDB2*	0.87	3.7×10^−5^	2.62	4.9×10^−6^	/	/
		*MYBPC3*	−2.27	7.0×10^−28^	−6.83	2.1×10^−35^	−6.16	5.6×10^−31^
		*SPI1*	−1.72	2.0×10^−16^	−5.21	1.7×10^−20^	−4.32	2.3×10^−16^
			−0.73	5.8×10^−4^	−5.20	3.8×10^−20^	−5.43	3.8×10^−24^
	cg01199135	*SDK2*	−1.98	1.9×10^−5^	−2.82	7.2×10^−7^	−0.63	2.8×10^−1^
	cg03455424	*FLJ33590*	1.14	1.2×10^−5^	0.97	3.8×10^−3^	/	/
	cg09786634	*RPTOR*	1.97	5.4×10^−9^	2.82	1.4×10^−5^	2.71	2.4×10^−5^
	cg10833299	*FLJ33590*	0.89	1.4×10^−4^	0.66	3.7×10^−2^	/	/
	cg11949518	*CHMP6*	1.05	1.1×10^−10^	1.69	2.4×10^−5^	0.87	2.8×10^−2^
		*FLJ90757*	1.16	8.4×10^−13^	1.10	5.6×10^−3^	0.80	3.9×10^−2^
		*RPTOR*	0.81	6.4×10^−7^	1.21	2.6×10^−3^	0.70	8.4×10^−2^
	cg14750269	*PNMAL1*	−3.61	7.9×10^−15^	−4.78	1.2×10^−5^	−3.28	4.0×10^−3^
	cg18948877	*FLJ33590*	−2.25	3.8×10^−6^	−3.45	1.1×10^−9^	/	/
	cg23194629	*DHX33*	1.31	1.5×10^−4^	2.79	1.6×10^−5^	/	/
		*RABEP1*	6.29	1.6×10^−83^	3.66	3.5×10^−12^	3.65	2.2×10^−12^
		*RPAIN*	−1.20	5.5*10^−4^	9.01	1.0*10^−47^	/	/
			1.35	9.4×10^−5^	3.45	8.3×10^−8^	/	/
	cg26207766	*FLJ33590*	0.82	4.7×10^−6^	0.67	2.3×10^−3^	/	/
B	DMR	Gene	Beta	P value	Beta	P value	Beta	P value
	2q37	*FLJ33590*	2.63	1.0×10^−4^	1.97	2.7×10^−2^	/	/
	19q13	*ZNF134*	−3.17	2.3×10^−7^	−3.15	1.9×10^−6^	/	/
		*ZNF154*	−8.87	5.7×10^−51^	−8.50	1.9×10^−40^	−7.96	4.1×10^−37^
		*ZNF211*	−3.74	9.1×10^−10^	−4.03	9.3×10^−10^	−4.21	6.2×10^−11^
		*ZNF586*	−2.23	2.8×10^−4^	−2.38	3.3×10^−4^	/	/
		*ZNF671*	−6.18	1.4×10^−24^	−6.06	9.9×10^−21^	−5.65	1.8×10^−18^
		*ZNF776*	−3.65	2.4×10^−9^	−3.36	3.7×10^−7^	/	/
	22q13	*LGALS1*	2.64	5.3×10^−4^	3.29	1.9×10^−4^	3.61	3.6×10^−5^
		*NOL12*	−2.85	1.8×10^−4^	−2.28	9.8×10^−3^	/	/
		*PDXP*	2.95	1.1×10^−4^	2.79	1.6×10^−3^	/	/
		*TRIOBP*	−5.97	2.8×10^−15^	−3.51	3.2×10^−5^	−2.80	6.8×10^−4^
	6p21	*NOTCH4*	−3.83	1.2×10^−4^	−2.85	2.5×10^−2^	/	/

Three regression models were used to identify associations between DNA methylation at DMPs (A) and DMRs (B) with proximal gene expression. The first model contained no covariates, while the second model contained only the respective meQTL (methylation quantitative trait locus) as a covariate and the third model included both meQTL and eQTL (expression QTL). Only the results for associations that were significant (*p* < 0.001) in the first model and nominally significant in the second model are shown (see Table S6 for full results). Missing values for the third column are due to lack of an identifiable eQTL.

As with the DMPs, we investigated whether methylation at the DMRs associates with proximal gene expression. Results show that the methylation at six out of the seven DMRs associates with at least one transcript without adjusting with genetics (Table S6). After adjusting with the respective meQTL, the association between 4 DMRs and 15 transcripts retained nominal statistical significance (Table 4B). Further adjusting the model with both meQTL and eQTL did not drastically affect the statistical significance of the results.

### Association of DNA methylation at DMPs and DMRs with cardiometabolic phenotypes

Next, we assessed whether the differential methylation between Eastern and Western Finns could potentially affect cardiometabolic health. Out of the 21 DMPs tested, we found associations with CMD risk phenotypes, independent of genetics, for 5 DMPs and 4 DMRs (Table 5). Associating CMD-risk phenotypes include triglycerides, glucose, total cholesterol and LDL cholesterol, as well as insulin treatment and a metabolic syndrome diagnosis following harmonized criteria [57]. The full results of nominally significant associations for DMPs and DMRs before and after adjusting with genetics can be found in Table S7.

**Table 5.** Association of methylation at DMPs and DMRs with cmd-risk phenotypes.

A	CpG	Gene Name	Phenotype	Beta	CI2.5	CI97.5	SE	P value
	cg03455424	/	LDL cholesterol	0.82	0.15	1.48	0.34	1.6×10^−2^
			Total cholesterol	0.80	0.14	1.45	0.33	1.7×10^−2^
	cg11472901	/	Glucose	−1.29	−2.14	−0.45	0.43	2.8×10^−3^
			Hypercholesterolemia medication	−4.85	−9.51	−0.23	2.36	4.0×10^−2^
			Metabolic syndrome	−3.96	−6.82	−1.13	1.45	6.3×10^−3^
			Triglycerides	−1.24	−2.04	−0.43	0.41	2.6×10^−3^
	cg18948877	*LINC01237*	Glucose	−1.30	−2.35	−0.25	0.53	1.5×10^−2^
	cg26740318	*LDHAL6A*	Insulin treatment	13.84	1.91	27.40	6.45	3.2×10^−2^
	cg27489373	*CLCN7*	Apolipoprotein B	0.88	0.29	1.47	0.30	3.6×10^−3^
			Triglycerides	1.07	0.49	1.64	0.29	2.9×10^−4^
B	DMR	Gene Name	Phenotype	Beta	CI2.5	CI97.5	SE	P value
	11p15	*LDHAL6A*	Insulin treatment	29.54	6.51	56.72	12.79	2.1×10^−2^
			Systolic blood pressure	−1.06	−2.07	−0.06	0.51	3.8×10^−2^
	19q13	*ZNF551, AC003006.7, ZNF154*	Metabolic syndrome	−5.61	−9.77	−1.55	2.09	7.4×10^−3^
	22q13	*NOL12, TRIOBP*	Triglycerides	1.69	0.20	3.17	0.76	2.6×10^−2^
	6p21	*PPT2, PRRT1*	Lipoprotein(a)	2.57	0.07	5.07	1.28	4.4×10^−2^

The associations between methylation at DMPs (A) and DMRs (B) and CMD-related phenotype were adjusted with sex, age, BMI, smoking, fasting status and meQTL (methylation Quantitative Trait Locus). Only the nominally significant results after adjusting with meQTL are shown in this table, see Table S7 for full results. LDL cholesterol, Total cholesterol, Glucose and Triglycerides were measured in mmol/l. Apolipoprotein B was measured in g/l. Systolic blood pressure was measured in mmHg. Lipoprotein (a) was measured in mg/l.

Since the YFS cohort is a population cohort consisting of relatively young and healthy individuals (34–48 y old in 2011), most participants had not yet started showing severe CMD phenotypes and so, effects of altered DNA methylation patterns may not be seen. We therefore replicated the analysis of association between methylation and CMD risk phenotype in LURIC, a cardiovascular patient cohort from Germany. Out of the 21 DMPs and 7 DMRs tested, 8 DMPs and 3 DMRs associated with one or more CMD-risk phenotype independently of genetics (Table 6). Full results before and after adjusting with genetics can be found in Table S8. Numerous loci associated with triglyceride, HbA1c and cholesterol levels as well as fasting insulin (*p* < 0.05). Methylation at both cg26740318 and DMR_11p15, in which the CpG site lies, associates with fasting insulin levels. Interestingly, cg11472901, cg18948877 (located in the *LINC01237* gene body) and cg26740318 (located in the *LDHAL6A* TSS1500 region) as well as DMR_11p15, DMR_19q13 and DMR_22q13 associate with phenotypes in both YFS and LURIC. Of these, associating phenotypes between cohorts is remarkably similar for cg26740318 and DMR_11p15 which associate with insulin treatment in YFS and fasting insulin levels in LURIC and DMR_22q13 which associates with triglyceride levels in both cohorts.

**Table 6.** Association of methylation at DMPs and DMRs with cmd-risk phenotypes in LURIC.

A	CpG	Gene Name	Phenotype	Beta	CI2.5	CI97.5	SE	P value
	cg00674440	*MADD*	Triglycerides	0.92	0.46	1.39	0.24	1.2×10^−4^
	cg10619644	*AUTS2*	HbA1c	0.91	0.25	1.56	0.33	6.6×10^−3^
	cg11472901	*/*	Venous thrombosis	2.39	0.13	4.67	1.16	3.9×10^−2^
	cg13902335	*MREG*	Fasting insulin	−0.78	−1.53	−0.03	0.38	4.2×10^−2^
			HbA1c	−1.18	−1.95	−0.40	0.40	3.1×10^−3^
	cg15720821	*/*	Triglycerides	0.71	0.13	1.28	0.29	1.6×10^−2^
	cg18948877	*LINC01237*	Diastolic blood pressure	−1.39	−2.48	−0.31	0.55	1.2×10^−2^
			Total cholesterol	−1.38	−2.47	−0.29	0.55	1.3×10^−2^
			Triglycerides	−1.49	−2.56	−0.43	0.54	6.1×10^−3^
	cg23194629	*NUP88*	Total cholesterol	0.58	0.02	1.14	0.29	4.4×10^−2^
			Triglycerides	1.30	0.76	1.85	0.28	3.1×10^−6^
	cg26740318	*LDHAL6A*	Fasting insulin	−0.51	−0.86	−0.17	0.18	3.6×10^−3^
			Triglycerides	−0.59	−0.95	−0.23	0.18	1.4×10^−3^
B	DMR	Gene Name	Phenotype	Beta	CI2.5	CI97.5	SE	P Value
	11p15	*LDHAL6A*	Fasting insulin	−0.53	−1.03	−0.02	0.26	4.0×10^−2^
	19q13	*ZNF551, AC003006.7, ZNF154*	LDL cholesterol	1.11	0.30	1.92	0.41	7.3×10^−3^
	22q13	*NOL12, TRIOBP*	Triglycerides	1.47	0.56	2.38	0.47	1.6×10^−3^
			LDL cholesterol	−1.25	−2.18	−0.32	0.47	8.5×10^−3^

The associations between methylation at DMPs (A) and DMRs (B) and CMD-related phenotype in LURIC, a German cardiovascular patient cohort. Regression models were adjusted with sex, age, BMI, smoking and meQTL (methylation Quantitative Trait Locus). Only the nominally significant results after adjusting with meQTL are shown in this table, see Table S8 for full results. LDL cholesterol, Total cholesterol and Triglycerides were measured in mg/dL. Diastolic blood pressure was measured in mmHg Fasting insulin measurements are in U/l. HbA1c measurements are in %.

## Discussion

We have shown for the first time that East- and West-originating Finns exhibit distinct DNA methylation profiles. We identified 21 DMPs (FDR <0.05 and Δβ >2.5%), as well as 7 DMRs between Finns originating from Eastern or Western regions. Furthermore, we have exploited the multiple omics available in the YFS cohort to gain insight to the establishment and potential function of the methylation at these DMPs and DMRs. All the identified DMPs and DMRs have a strong genetic basis; however, methylation at 13 DMPs may also be partly influenced by other factors. Independently of genetics, methylation at 11 DMPs and 4 DMRs associates with gene expression and 5 DMPs and 4 DMRs with cardiometabolic phenotypes in YFS. Similarly, methylation at 8 DMPs and 3 DMRs associates with cardiometabolic phenotypes independently of genetics in LURIC, a German cardiovascular patient cohort, with the association replicating for cg26740318, DMR_11p15 and DMR_22q13 across cohorts.

In our study setting, we used the participant’s grandparental birthplace to define their Eastern or Western origins. Since internal migration in Finland was very limited before the mid-1900s [59], the grandparental birthplace of our participants (aged 30–46 in 2011) is indicative of a long family history in the same region. Limited internal migration over a long period of time most likely resulted in genetic drift which led to the observed genetic discrepancy between the sub-populations [3,60–63]. These findings are also reflected in the YFS cohort genetic data, whose greatest source of variation can be explained by the East/West origins of the participants.

Among the most notable environmental differences between North-Eastern and South-Western Finnish regions are differences in socio-economic status and urbanization [64–66]. Both socio-economic status and urbanization have been associated with cardiometabolic health outcomes [64,67,68] and DNA methylation has been proposed as the molecular mediator for this environmental effect on health [69–71]. For example, lower education attainment is associated with both increased cardiovascular disease [72] and increased epigenetic age [71], although the exact role of the epigenetic changes remains unclear. The difference in socio-economic status and urbanization could be potential causes for the genetic independent observed differences in DNA methylation between East- and West-originating identified in this study.

The identified DMPs are located in or near 10 protein-coding genes and in one long non-coding RNA (*LINC01237*) with notable clustering in chromosomes 2 (6 DMPs) and 17 (6 DMPs). The locus with the highest statistical difference, in addition to more than 5% difference in the methylation β, between East- and West-originating Finns was cg15470645, located in the TSS1500 of *NEU4*, where another identified DMP (cg10833299) is also located. Both these CpGs are also within DMR_2q37, which was found to be hyper-methylated in the population originating from Eastern Finland. *NEU4* encodes a sialidase which has been implicated in cancer, lysosomal storage disease, neurogenesis and apoptosis [73] and intriguingly, methylation at this region has been associated with prenatal events [74,75].

Similarly, the methylation at two DMPs (cg09786634 and cg11949518) in the *RPTOR* gene body was significantly down-regulated in Eastern Finns compared to Western Finns. This is particularly interesting as *RPTOR* encodes for raptor (regulatory associated protein of m*TOR*), which is an integral part of the mTOR complex 1 [76,77], known to be involved in numerous important cellular processes including cell growth, metabolism, lysosome biogenesis and autophagy [78]. Previous research has identified altered DNA methylation patterns in *RPTOR* in lung cancer [79] and breast cancer [80,81] patients, although some of these results failed to replicate [82]. Mouse models have also shown that mTOR1 inhibition increases lifespan and improves cardiac function in a disease background [83]. A previous study identified three other CpGs in the same region which are hypermethylated in atherosclerotic aorta samples [84].

Out of the seven DMRs identified in this study, DMR_6p22 and DMR_19q13 overlap with genes encoding Krüppel-associated box (KRAB) domain containing zinc finger proteins. DMR_6p22 overlaps with *ZFP57*, which is important for the maintenance of methylation in imprinting control regions [85–87]. A previous study found that maternal folate intake during pregnancy associates with the methylation of a region that largely overlaps the DMR_6p22 region identified in this study [88]. On the other hand, DMR_19q13 overlaps with *ZNF551* and *ZNF154*, and DNA methylation in three CpGs in *ZNF154* previously showed a positive correlation with age [89]. In our data, methylation at DMR_19q13 also associates strongly with the expression of multiple zinc finger proteins, which have many diverse functions including transcriptional regulation, post-transcriptional modifications and protein–protein interactions [90]. Several zinc finger proteins play a role in cardiometabolic health and have been implicated in lipid metabolism [91,92], diabetes [93–95] and congenital heart diseases [96–98].

In addition to differences in DMPs and DMRs, a 0.02 increase in epigenetic age was identified in Eastern men when compared to their Western counterparts using the DunedinPACE metric but not with any of the other epigenetic age clocks included in this analysis. The DunedingPACE clock is the only clock trained on a longitudinal data and it yields a measure of the pace of ageing [53] rather than the biological age, making it unique from the other epigenetic age measures. A value of 1 in DunedinPACE indicates biological ageing of 1 y within a calendar year and thus, a higher pace of ageing is associated with a faster decline of physical and cognitive health [99,100]. Our finding is in line with other literature suggesting that Eastern Finns are worse off health-wise than Western Finns [5,11,12].

The mechanistic origin of DNA methylation differences between Eastern and Western Finns is made complicated due to the dual effects of genetic and environmental factors on the establishment of DNA methylation levels. Differential methylation between the two populations could be caused by different distribution of meQTLs across the sub-populations, as was previously reported in studies among genetically diverse populations [34,35]. The proposed mechanism of *cis*-meQTL action is through the disruption of CCTC-binding factor or transcription factor binding which then affects nearby DNA methylation [101]. Multiple *cis*-meQTLs were identified for each DMP and DMR, which can probably be attributed to linkage disequilibrium which causes nearby loci to be jointly inherited. However, since some loci remained statistically differentially methylated after adjusting with the top meQTL, a combination of environmental and genetic factors could be at play, although the combined effect of multiple meQTLs, *trans*-meQTLs or other confounders cannot be excluded.

Investigating the association between DNA methylation and proximal gene expression gives insight to the potentially functionally relevant loci. Among the most statistically significant associations in the gene expression analysis is that of DNA methylation at cg00674440, located in the *MADD* gene body, and the expression of *MYBPC3* and *SPI1* transcripts. MYBPC3 is a sarcomere component of heart muscle and mutations in the gene are associated with hypertrophic cardiomyopathy [102], while SPI1 is a transcription factor with an ETS-domain and its expression in cardiac tissue was found to increase during myocardial infarction as a result of reduced DNA methylation, in mice [103]. A strong association was also identified between methylation at cg23194629, located in the gene body of *NUP88*, and *RABEP1* expression. *RABEP1* is involved in vesicular transport and autophagy [104] and one study has found its increased expression in heart and lung tissue to be associated pulmonary arterial hypertension in mice [105].

By looking at the association between methylation at DMPs and DMRs and cardiometabolic disease risk phenotypes, we can gain insight to the potential role of DNA methylation in the observed East/West discrepancy of CMDs. For this analysis, we also utilized the LURIC cohort since it consists of patients diagnosed with cardiometabolic diseases who have more severe phenotypes than the YFS cohort. Comparing the results from YFS and LURIC, DNA methylation at cg11472901, cg18948877, cg26740318, DMR_11p15, DMR_19q13, and DMR_22q13 associated with various CMD-risk related phenotypes in both cohorts. Replication across both cohorts supports the hypothesis that altered methylation levels at these positions and regions may impact cardiometabolic health. Interestingly, associations observed for cg26740318, DMR_11p15 and DMR_22q13 replicate closely between the two cohorts.

Increased methylation at cg26740318, located in the TSS1500 region of *LDHAL6A*, was associated with increased insulin treatment in YFS and decreased fasting insulin levels in LURIC. These findings were also similar for methylation at DMR_11p15 which overlaps the cg26740318 locus. Higher methylation at both the DMP and DMR was found in East-originating Finns, which aligns with statistics that show that the incidence of type II diabetes is higher in north-eastern regions of Finland [106]. The LDHAL6A protein is a lactate dehydrogenase which is primarily expressed in the testes [107]; however, it was also found to be differentially expressed in pancreatic β cells of patients with type II diabetes [108].

Our data also revealed that increased DNA methylation at DMR_22q13 associates with elevated triglyceride levels in both YFS and LURIC. Higher methylation levels in this region were found in East-originating Finns, again suggesting that they are metabolically worse off. Interestingly, a previous study identified an association between increased galactin-1 levels, encoded by *LSGAL1* whose expression was found to be associated with DMR_22q13 methylation in this study, with increased triglycerides in humans [109]. Galectin-1 is a β-galactoside binding protein with functions in metabolism and inflammation and it has been linked to obesity and type II diabetes [110,111].

Out of the 21 DMPs identified between East and West-originating Finns, previous literature revealed association with CMD phenotypes at two other loci. DNA methylation at cg08946995 in whole blood was linked to coronary artery disease [112], while methylation at cg26207766 in liver biopsy samples was linked to obesity and type II diabetes [113]. These two sites did not associate with phenotypes in our main population analysis; however, this can partly be due to our DNA methylation data originating from blood rather than liver samples. A list of the main studies that have previously reported an association between any of the 21 DMPs and any phenotypes can be found in Table S9.

## Strength and limitations of the study

The multi-omic approach of this study is a considerable strength. Single-omic studies are easier to perform and interpret; however, they can provide limited insight into the mechanisms of complex diseases due to the interplay of different omics [114]. The bioinformatic analysis of multi-omic data allows for more accurate elucidation of the molecular mechanisms involved in CMD pathophysiology as well as the development of personalised treatment [115–117]. In our analysis, we integrated methylome, genome and transcriptome data, as well as blood measurements for CMD-related traits, for a more holistic investigation of the establishment and effect of DNA methylation. Our findings highlight the particular importance of taking the genetic component into consideration, particularly when studying a population with known genetic sub-structure, as we determined that all the DMPs and DMRs have a strong genetic basis. If genetic data were not available, online databases documenting known associations between DNA methylation and SNPs, such as the Genetics of DNA methylation consortium [118], can be used. The availability of genetic data in our case, however, also allowed us to account for the genetic factors in the transcriptomic and phenotypic analysis, in order to minimize their relative effect on the results and, as much as possible, isolate the environmental effects.

A significant concern related to DNA methylation measurements with available array technology is the poor reproducibility at specific probes [119–121]. The reliability of 10 probes out of the 21 sites identified in this study was included in a previously published reliability measurement study where the same samples were analysed with both 450K and EPIC arrays [119]. All 10 probes were found to have a reliability of ≥75%, which is considered highly reliable and therefore suggests our results may be reproducible. Replication in a different cohort, using different methodology and sequencing techniques, would be required to confirm our results.

## Conclusions

Taken together, the main results indicate that some of the differentially methylated loci between East- and West-Finns may be functionally active in modulating transcription and may be contributing to the CMD difference between Eastern and Western Finns. Our analysis has exploited the availability of multi-omic data to account for the genetic effect so that our results will more accurately reflect the extrinsic, rather than intrinsic, influence on DNA methylation levels.

## Supplementary Material

Supplementary Tables.xlsx

Supplementary Figures.docx

## Data Availability

Data used in this study are considered sensitive due to potential identifiability of participants. Data sharing is therefore restricted under the regulations on professional secrecy (Act on the Openness of Government Activities, 612/1999) and on sensitive personal data (Personal Data Act, 523/1999, implementing the EU data protection directive 95/46/EC). Due to these legal restrictions, the data from this study cannot be stored in public repositories or otherwise made publicly available. The data are available from the authors upon reasonable request.
