# Supplementary material for: Identification and functional characterisation of DNA methylation differences between East- and West-originating Finns
Source: Epigenetics. 2024 Sep 1;19(1):2397297. doi: 10.1080/15592294.2024.2397297 (PMC11382697; doi:10.1080/15592294.2024.2397297)
Supplement: Supplementary Figures.docx [file KEPI_A_2397297_SM0032.docx]

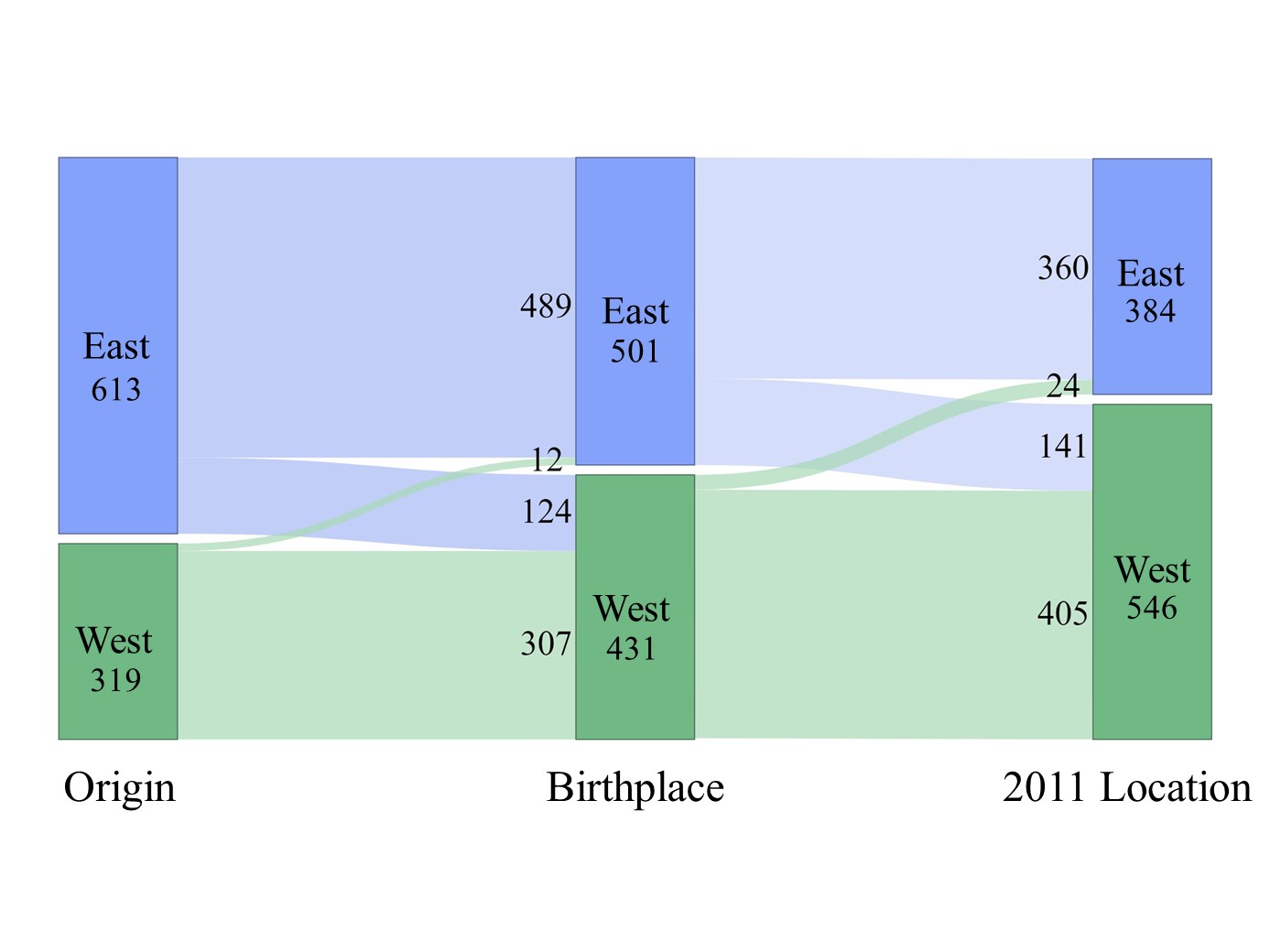


**Fig** **S1. Study population’s origin, birthplace and living location in 2011.** A timeline of the study population at three timepoints: their grandparental birthplace (origin), the participant’s own birthplace and the reported living location in 2011 when the DNA methylation data was collected. Only participants with data at all 3 timepoints were included in this analysis (n=930).


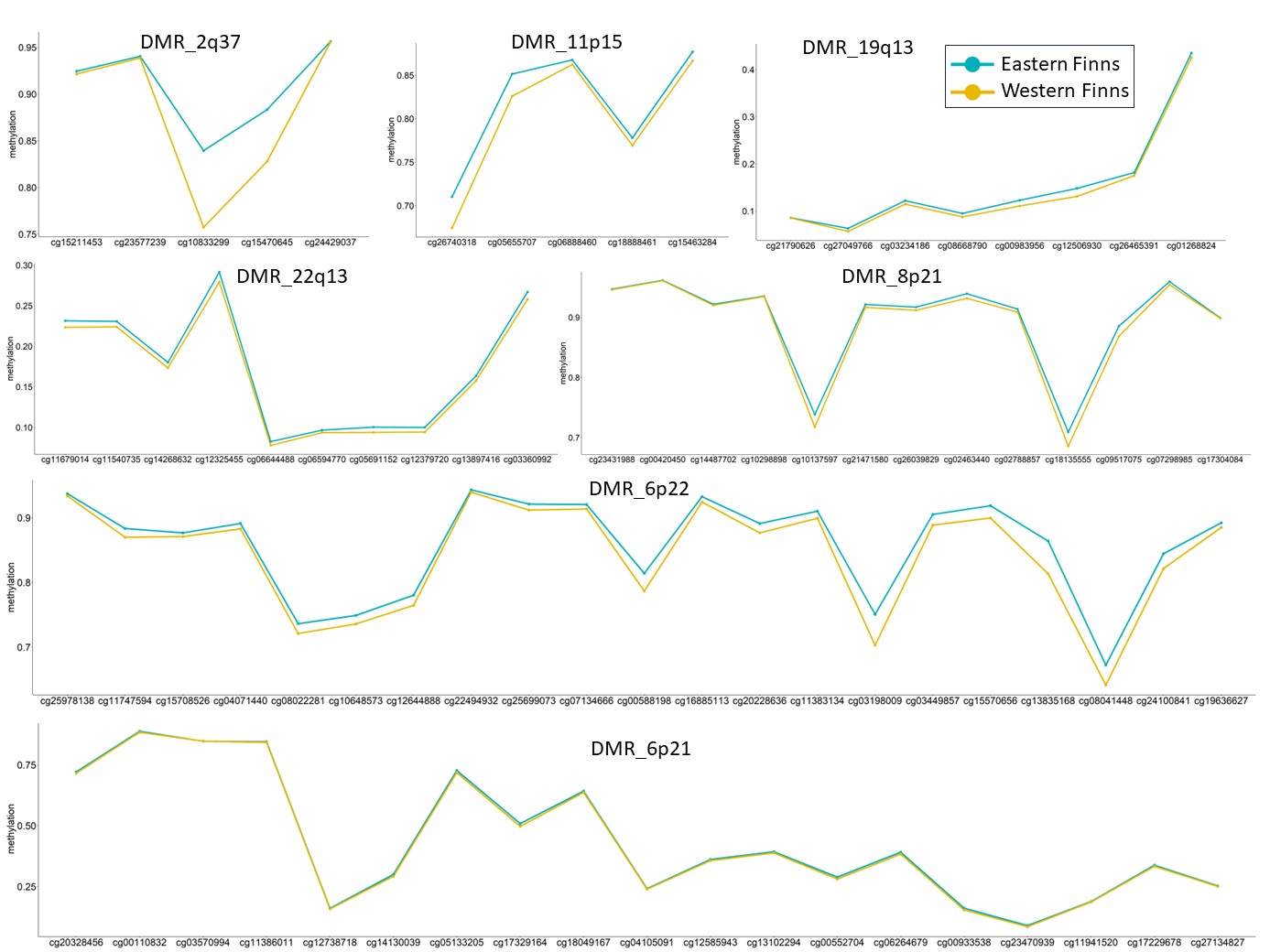


**Fig S2. Differentially Methylated Regions between Eastern and Western Finns.** Differentially methylated regions (DMRs) between Eastern- and Western-originating Finns (n=969) were identified using the DMRcate R package. The mean methylation value at each CpG site for each group was plotted. The CpG sites in each DMR are ordered according to genomic location.


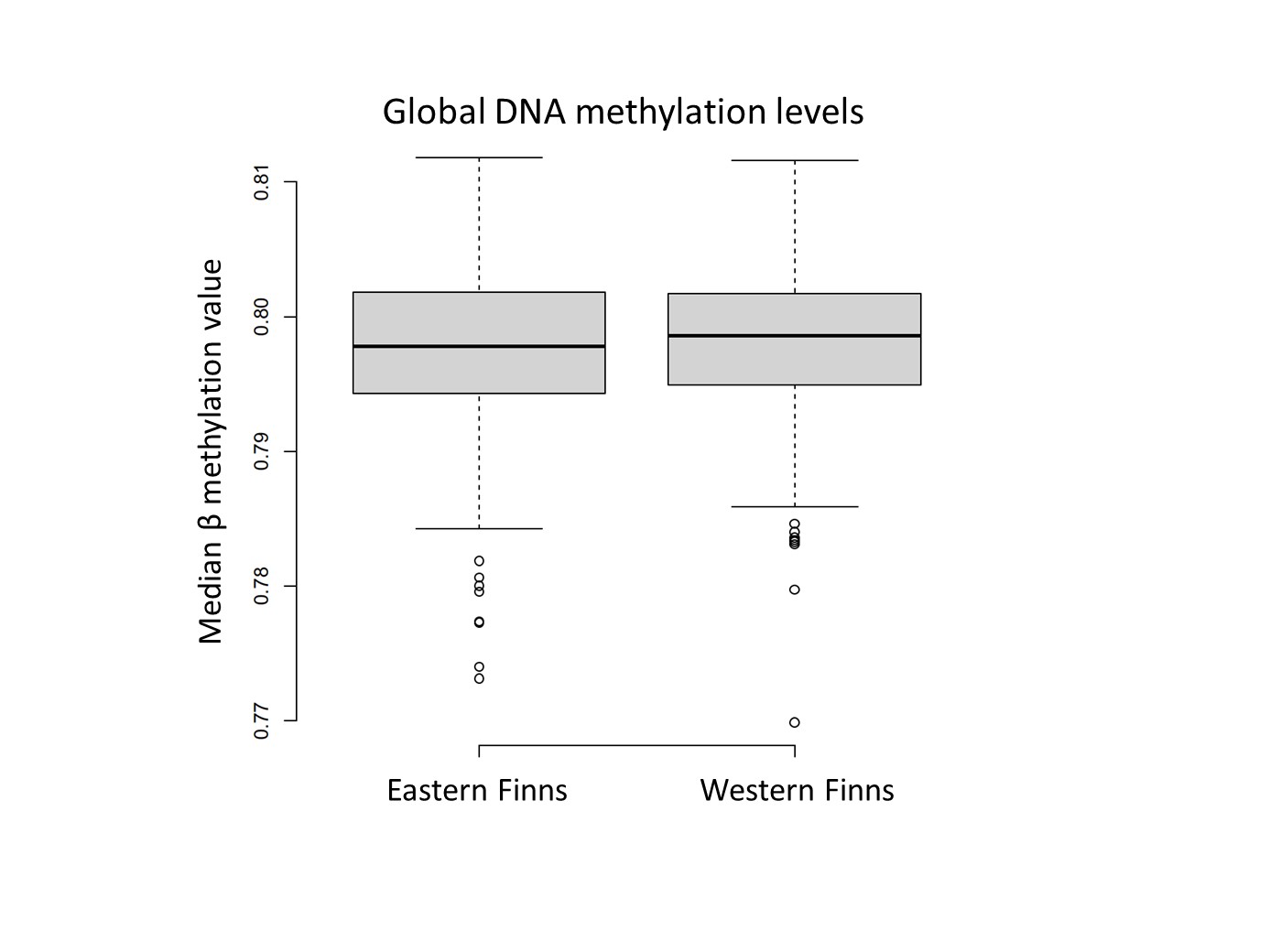


**Fig S3. No Difference in global methylation between Eastern and Western Finns.** Median methylation Beta values between Eastern- and Western-originating Finns (n=969) were compared by linear regression. The regression model was adjusted with age, sex, BMI, smoking, blood cell composition and the first 30 principal components of the technical probes and the methylation levels were inverse-normal transformed. No statistically significant difference was identified between the two groups (p = 0.12).
